# Transcriptional regulation of photoprotection in dark-to-light transition—More than just a matter of excess light energy

**DOI:** 10.1126/sciadv.abn1832

**Published:** 2022-06-03

**Authors:** Petra Redekop, Emanuel Sanz-Luque, Yizhong Yuan, Gaelle Villain, Dimitris Petroutsos, Arthur R. Grossman

**Affiliations:** 1 Department of Plant Biology, The Carnegie Institution for Science, 260 Panama St, Stanford, CA 94305, USA.; 2 Department of Biochemistry and Molecular Biology, University of Cordoba, 14071 Cordoba, Spain.; 3 Université Grenoble Alpes, CNRS, CEA, INRAe, IRIG-LPCV, 38000 Grenoble, France.; 4 Department of Biology, Stanford University, Stanford, CA 94305, USA.

## Abstract

In nature, photosynthetic organisms are exposed to different light spectra and intensities depending on the time of day and atmospheric and environmental conditions. When photosynthetic cells absorb excess light, they induce nonphotochemical quenching to avoid photodamage and trigger expression of “photoprotective” genes. In this work, we used the green alga *Chlamydomonas reinhardtii* to assess the impact of light intensity, light quality, photosynthetic electron transport, and carbon dioxide on induction of the photoprotective genes (*LHCSR1*, *LHCSR3*, and *PSBS*) during dark-to-light transitions. Induction (mRNA accumulation) occurred at very low light intensity and was independently modulated by blue and ultraviolet B radiation through specific photoreceptors; only *LHCSR3* was strongly controlled by carbon dioxide levels through a putative enhancer function of CIA5, a transcription factor that controls genes of the carbon concentrating mechanism. We propose a model that integrates inputs of independent signaling pathways and how they may help the cells anticipate diel conditions and survive in a dynamic light environment.

## Teaser

Light, photoreceptors, CO_2_, and global regulatory elements integrate on the expression of genes critical for photoprotection.

## INTRODUCTION

Light absorption and its conversion into chemical energy by photosynthetic organisms is an essential process for almost all life on our planet. Photosynthetic organisms have evolved to efficiently capture light energy when the intensity is low and quench absorbed excitation energy when it exceeds the photon flux density needed to saturate photosynthetic electron transport (PET). Excess light leads to the generation of reactive oxygen species that cause cellular damage and even cell death. Photoprotection requires the activities of a set of proteins that functions to dissipate excess absorbed light energy before it is used to drive reaction center function. In the green alga *Chlamydomonas reinhardtii* (Chlamydomonas throughout), the light harvesting complex stress-related proteins (LHCSR3) (encoded by *LHCSR3.1* and *LHCSR3.2*, which only differ slightly in their promoters) and LHCSR1, and the photosystem II subunit S protein (PSBS) (encoded by *PSBS1* and *PSBS2*; the proteins differ by one amino acid in their signal peptide) are often described as photoprotective proteins that accumulate in response to high light (HL) and ultraviolet B (UV-B) radiation (280 to 315 nm) (*1*–*7*). These photoprotective proteins are critical for rapid nonphotochemical quenching (NPQ) of excess absorbed light energy through a thermal dissipation process designated energy-dependent quenching (qE). For the Chlamydomonas *PSBS* genes, the transcript and proteins accumulate transiently in response to HL and UV-B radiation (*4*, *6*, *7*). The exact function of PSBS in Chlamydomonas needs further elucidation, although it was found to positively affect acclimation to HL (*8*), and studies in vascular plants have demonstrated that it functions in conjunction with the xanthophyll cycle and a ΔpH across the thylakoid membranes to elicit qE (*1*, *9*, *10*). LHCSR proteins (LHCSX in diatoms) are the dominant “quenching” proteins in algae, and while present in moss, no orthologs have been identified in vascular plants (*11*–*14*).

To elicit an efficient photoprotection response, cells would need to accumulate the photoprotective proteins before or very soon after exposure to HL. At the transcriptional level, *LHCSR* and *PSBS* genes are strongly induced during the dark to light transitions, especially when this transition is abrupt (*15*). However, the signals that prime cells for eliciting photoprotective processes are still not well understood. There are many questions concerning the mechanisms and the factors controlling accumulation of *LHCSR3*, *LHCSR1*, and *PSBS* transcripts and the encoded proteins. Expression of these genes is affected by specific photoreceptors including the UV RESISTANCE LOCUS 8 (UVR8) (*7*, *16*) and the blue light photoreceptor phototropin (PHOT) (*17*, *18*).

UVR8 in *Arabidopsis thaliana* is homodimeric and absorbs UV-B radiation through tryptophan residues with a peak in its action spectrum at 260 to 280 nm (*19*). The absorption of UV-B radiation by UVR8 causes monomerization of the photoreceptor and facilitates its interactions with CONSTITUTIVELY PHOTOMORPHOGENIC 1 (COP1), a protein with E3 ubiquitin ligase activity (*20*, *21*), and SUPPRESSOR OF PHYA-105 1 (SPA1) (*22*). This complex prevents degradation of ELONGATED HYPOCOTYL 5, a transcription factor that activates gene expression in response to UV-B radiation (*23*), including the responses associated with acclimation of plants to excess absorbed excitation energy (*24*).

Chlamydomonas UVR8, COP1, and SPA1 orthologs also have functions related to quenching excess absorbed light energy (*16*, *25*). COP1 and SPA1, along with CULLIN4 and DAMAGED DNA BINDING 1, form an E3 ubiquitin ligase complex. This complex controls light-dependent transcription of Chlamydomonas *LHCSR* and *PSBS* genes and appears to be critical for suppressing expression of the genes associated with qE in low light (LL)–grown cells (*25*), which involves ubiquitination of a complex formed by two transcription factors, CONSTANS (Cr-CO) and the NUCLEAR FACTOR-Y (NF-Y). These factors are required for eliciting maximum induction of proteins associated with qE (*26*). The absorption of UV-B radiation by UVR8 leads to the interaction of monomeric UVR8 with COP1, which promotes SPA1/COP1 dissociation from Cr-CO, which, in turn, blocks Cr-CO ubiquitination and degradation and allows the formation of a stable Cr-CO/NF-Y transcription complex that elicits increased accumulation of *LHCSR* and *PSBS* transcripts (*26*, *27*).

The blue-light photoreceptor PHOT1 consists of two similar photosensory LOV1/2 (light-, oxygen-, and voltage-sensitive) domains at the N terminus and a serine/threonine kinase domain at the C terminus. Upon perception of blue light by LOV1/2, the photoreceptor is autophosphorylated and activates cellular responses that promote plant growth under weak light conditions. Higher plants code for two PHOTs, PHOT1 and PHOT2, with distinct and overlapping functions including phototropism, stomatal opening, chloroplast movement, and cotyledon and leaf expansion (*28*). In Chlamydomonas, there is a single PHOT (designated PHOT1) that controls expression of genes for enzymes in the chlorophyll and carotenoid biosynthesis pathways (*29*), regulates multiple steps of the sexual life cycle (*30*), and acts as light regulator of phototaxis (*31*). A link between PHOT1 and activation of the *LHCSR3* gene has also been established (*17*).

Recent evidence suggests that *LHCSR1*, *LHCSR3*, and *PSBS* in Chlamydomonas are transcribed in response to HL and UV-B radiation, with *LHCSR3* specifically requiring a PHOT-dependent and a light-dependent signal generated in the chloroplast that is still to be defined (*17*). *LHCSR1* (*6*) and *PSBS* (*32*) were previously proposed to be regulated by high white light by an unknown pathway. The work presented in this manuscript provides new insights into the features of radiation that affect the expression of these three photoprotective genes. We found that a strong induction of all three genes is observed following a shift from dark to LL; this LL-elicited increase in transcript abundances is mostly independent of PET but dependent on PHOT1. We also demonstrate that UV-B radiation independent of photosynthetically active radiation (PAR) can cause maximum accumulation of *LHCSR1*, *PSBS*, and near maximum accumulation of *LHCSR3* transcripts; this response is mostly suppressed in the *uvr8* mutant. Furthermore, *LHCSR3* is strongly regulated by CIA5 through a potential enhancer function that is needed to elevate expression under all conditions, while there is no or little CIA5-dependent control of *LHCSR1* or *PSBS* at the transcript level, although *PSBS* may be affected to a minor extent by high CO_2_. In addition, we discuss the potential integration of these signals in nature.

## RESULTS

### Photoprotective genes are induced at LL intensities

**Fig. 1.**
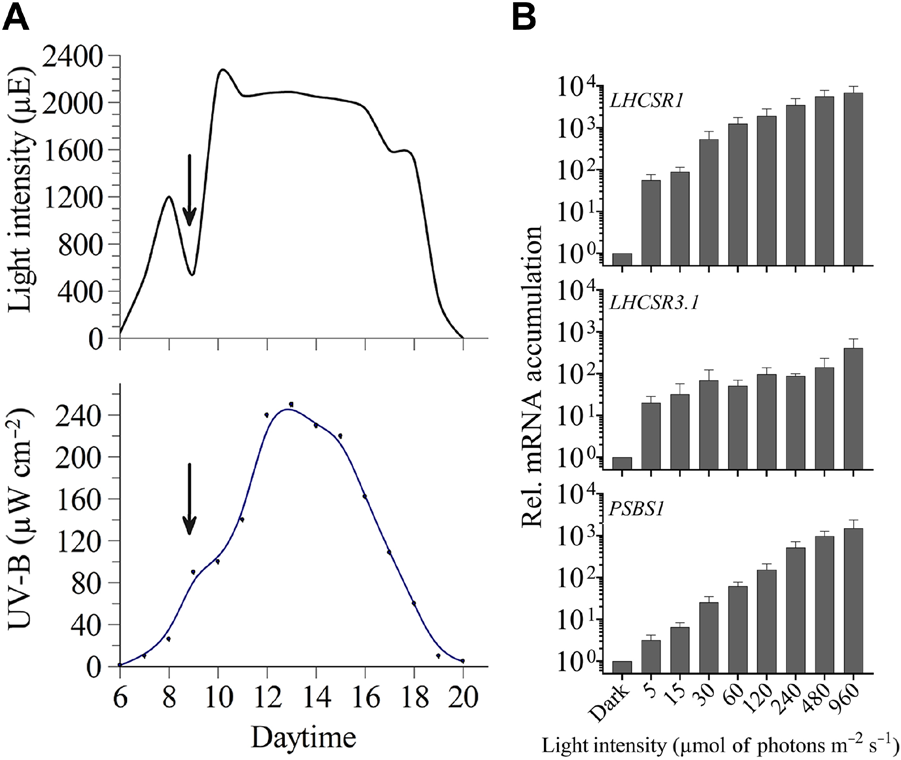
PAR and UV-B intensity from sunrise to sunset and changes in *LHCSR* and *PSBS1* transcript levels after irradiation at various light intensities. (**A**) The intensity of PAR and UV-B were monitored from dawn to dusk (July, California). (**B**) Accumulation of *LHCSR1*, *LHCSR3.1*, and *PSBS1* transcripts following incubation for 1 hour at different light intensities. WT CC-125 cells were grown in TAP at LL (30 μmol of photons m^−2^ s^−1^) and then transferred to the dark for 24 hours (in TAP, maintaining a fixed carbon source in the dark). Cells were then transferred to high salt medium (HSM; photoautotrophic conditions) and maintained for two additional hours in the dark (to reduce intracellular levels of acetate and bring cells into a physiologically relevant state in which they would most effectively quench excessive absorbed light) before a 1-hour light exposure at each of the indicated intensities. Abundances of each of the three transcripts were normalized to their dark control value. *n* = 3 + SD. Statistical analyses and *P* values are listed in data S1.

Light intensity and quality, including levels of UV-B, markedly change over the diel cycle. An hourly characterization of PAR and UV-B intensities was tracked from dawn to dusk in July in California under generally sunny skies, with some cloud cover at 9:00 to 10:00 a.m. Both PAR and UV-B intensities gradually increased, with a broad PAR peak reaching maximal values between 11:00 a.m. and 4:00 p.m. and a narrower UV-B peak (Fig. 1A). As shown in Fig. 1A (note arrow), clouds affect the intensity of PAR much more than that of UV-B radiation. Furthermore, from 6:00 to 8:00 a.m. the PAR intensity increased steeply from less than 100 μmol of photons m^−2^ s^−1^ to more than 1000 μmol of photons m^−2^ s^−1^ and then more gradually, reaching a peak of ~2000 μmol of photons m^−2^ s^−1^ that is sustained over a period of 5 to 6 hours. Hence, acclimation to HL occurs progressively, starting under very LL conditions in the early morning, with rapidly increasing intensities over the course of ~4 hours, reaching intensities that result in the hypersaturation of photosynthesis (photosynthesis saturates at ~400 to 1000 μmol of photons m^−2^ s^−1^) that is sustained over a large proportion of the day (*33*, *34*).

We postulated that algae accumulate transcripts from photoprotective genes (often described as “HL-responsive” genes) even in the early morning when there is a dark-to-light transition (when the light intensities are subsaturating). Levels of transcripts from *LHCSR1*, *LHCSR3.1*, and *PSBS1* genes were analyzed in dark-acclimated wild-type (WT) cells (the WT is CC-125, unless otherwise stated) after exposure to 1 hour of PAR at 5, 15, 30, 60, 120, 240, 480, and 960 μmol of photons m^−2^ s^−1^. The *LHCSR1* and *LHCSR3.1* genes showed strong induction even when exposed to very LL. There was a 56-fold increase for *LHCSR1* mRNA and a 20-fold increase for *LHCSR3.1* mRNA at 5 μmol of photons m^−2^ s^−1^ compared to cells maintained in the dark. Furthermore, although maximum levels of mRNA accumulation from these two genes were observed at the highest light intensities (960 μmol of photons m^−2^ s^−1^), it is remarkable that *LHCSR1* transcript levels showed a 500-fold induction after exposure to only 30 μmol of photons m^−2^ s^−1^, and *LHCSR3.1* exhibited an induction of >60-fold at 30 μmol of photons m^−2^ s^−1^. On the other hand, *PSBS1* was the least sensitive to low-intensity radiation (e.g., threefold at 5 μmol of photons m^−2^ s^−1^) and displayed a gradual and continuous increase in the level of its mRNA with increasing light intensity (Fig. 1B and fig. S1). The *LHCSR1* transcript also exhibited a gradual and continuous increase in transcript accumulation between 5 and 960 μmol of photons m^−2^ s^−1^ (~2 orders of magnitude; Fig. 1B and fig. S1). Of the three transcripts, *LHCSR3.1* exhibited the lowest additional increase in transcript accumulation following its initial sharp rise at 5 μmol of photons m^−2^ s^−1^; the difference between transcript abundance at 5 and 960 μmol of photons m^−2^ s^−1^ is ~20-fold. This increase was gradual from 5 to 480 μmol of photons m^−2^ s^−1^, with a further increase of ~2× between 480 and 960 μmol of photons m^−2^ s^−1^. In addition, we compared the levels of transcript accumulation across a light intensity gradient for the three genes from two different WT strains, CC-125 and CC-124 (fig. S1A), which demonstrated that, although similar patterns were observed, there were differences in the sensitivity of the two strains to light intensity, most likely the consequence of genetic differences between them (*35*).

### *LHCSR1*, *LHCSR3.1*, and *PSBS1* transcripts accumulate even when photosynthesis is blocked

**Fig. 2.**
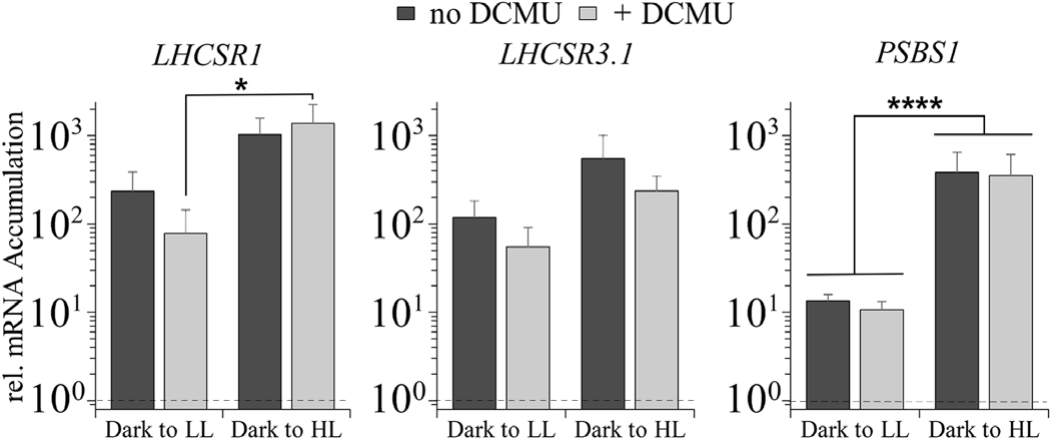
Abundances of *LHCSR1*, *LHCSR3.1*, and *PSBS1* transcripts after 1 hour in LL and HL, in the absence and presence of DCMU. WT CC-125, grown as described in the legend of Fig. 1, was exposed to either white LL (30 μmol of photons m^−2^ s^−1^) or HL (480 μmol of photons m^−2^ s^−1^) for 1 hour, in the absence or presence of 10 μM DCMU, which was added to the cultures immediately before light exposure. Data were normalized to 1 (shown as dashed line in graph), which was set as the initial dark level of the mRNA. There was no significant difference of transcript levels comparing the same samples treated with and without DCMU (although slight differences are seen in this graph). Accumulation of *PSBS1* transcripts between LL and HL was highly significant (*P* ≤ 0.0001), as well as the *LHCSR1* transcript in cells treated with DCMU in LL and HL (*P* = 0.0153). *n* = 3 + SD. There are no statistical differences for transcript accumulations within the same light treatment comparing different DCMU treatments. Error bars represent +SD, *n* ≥ 3. One-way analysis of variance (ANOVA) was performed. *P < 0.05 and ****P < 0.0001. See data S1 for all statistical analysis.

In previous works, it was concluded that the maximum accumulation of the LHCSR3 protein required active linear electron transport but was independent of the redox state of the plastoquinone (PQ) pool. This conclusion was supported by the findings that accumulation of the LHCSR3 protein was inhibited in the presence of DCMU [3-(3,4-dichlorophenyl)-1,1-dimethylurea], an inhibitor that blocks Q_b_ binding site of photosystem (PS) II and leads to oxidation of the PQ pool (*36*–*38*). The lack of LHCSR3 protein accumulation in DCMU-treated samples correlated with essentially no increase in the mRNA from *LHCSR3.1* and *LHCSR3.2* in cells transferred from LL to HL in the presence of DCMU (*17*, *39*). Despite this previous work suggesting that the linear PET is essential for *LHCSR3* induction, we examined the impact of DCMU on accumulation of the *LHCSR1*, *LHCSR3.1*, and *PSBS1* transcripts following a dark-to-light transition. Figure 2 shows that a transition from dark to LL (30 μmol of photons m^−2^ s^−1^ for 1 hour) strongly induces the three genes even in the presence of DCMU (Fig. 2). The *LHCSR3.1* transcript abundance increased 118- and 551-fold in LL and HL, respectively, and DCMU suppressed these increases by ~50%, indicating that, in the absence of linear electron flow, the cells can still induce expression of this gene by ~55- and 236-fold in LL and HL, respectively. For *LHCSR1*, DCMU caused a similar reduction in transcript accumulation in LL but not in HL. The absence of a significant DCMU-mediated effect on *LHCSR1* gene expression under HL agrees with previously published data (*39*). There was no effect of DCMU on *PSBS1* mRNA accumulation in either LL or HL, suggesting that *PSBS1* expression is completely independent of linear electron flow.

To elucidate the importance of the preacclimation conditions on induction of the photoprotective genes and explore differences between our results and those obtained previously (*17*, *38*), *LHCSR3.1* transcript levels were quantified in WT cells, either acclimated to the dark or LL (15 μmol of photons m^−2^ s^−1^) in high salt medium (HSM) overnight or transferred to HL (300 μmol of photons m^−2^ s^−1^) for 1 hour either in the presence or absence of DCMU. In the LLacclimated cells, transcript accumulation in HL was completely inhibited by the addition of DCMU (fig. S2) in agreement with previously reported results (*17*). However, after a preincubation in the dark, DCMU-treated cells exposed to HL exhibited an increase in the level of *LHCSR3.1* of 44-fold (fig. S2), which is in accord with the results presented in Fig. 2. These data highlight the impact of the preacclimation conditions (those under which cells are maintained before the test conditions) on transcript levels.

### PHOT1 regulates initial LL responses

**Fig. 3.**
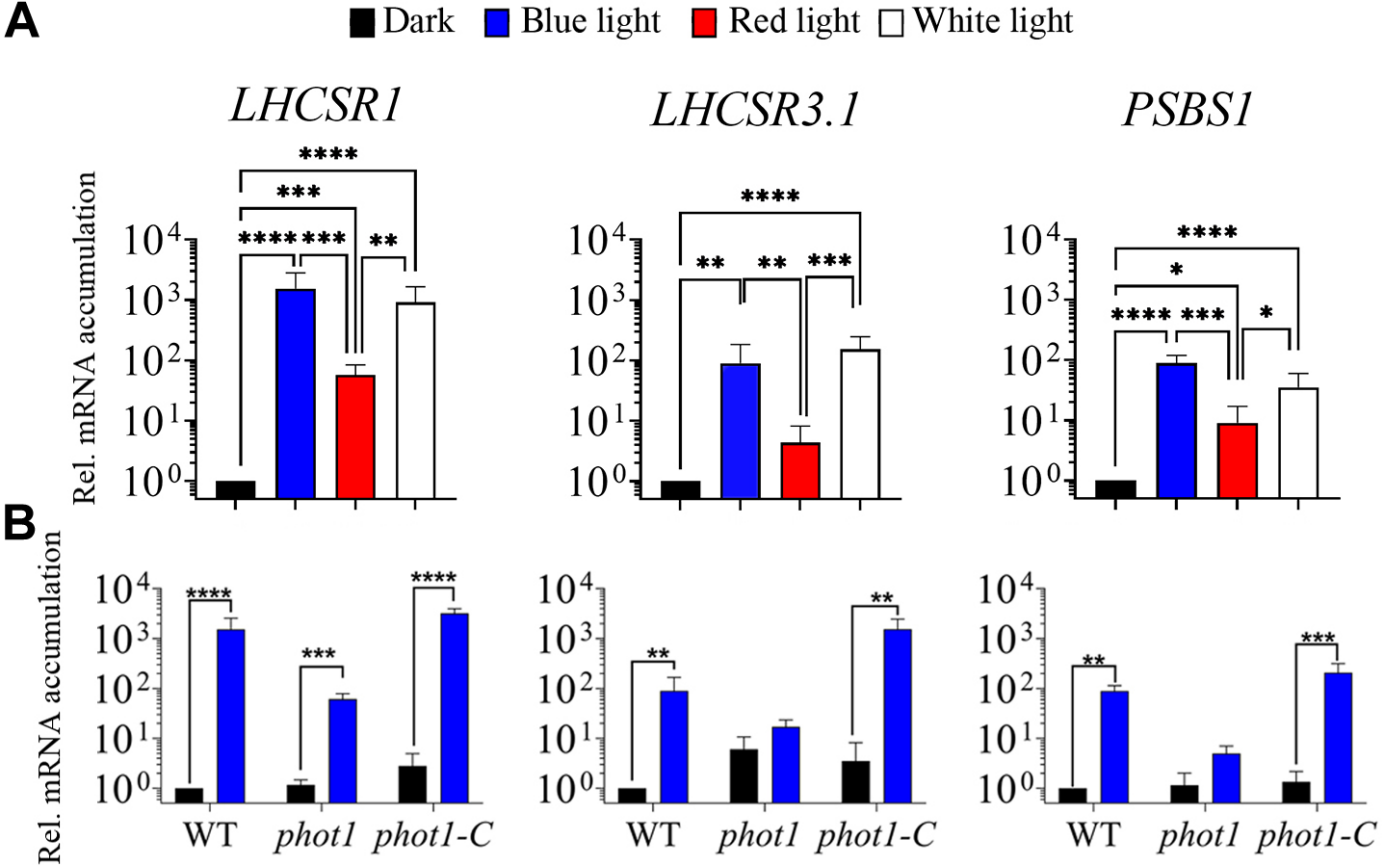
Transcript accumulation of *LHCSR1*, *LHCSR3.1*, and *PSBS1* after exposure to blue, red, or white light. WT (CC-125), *phot1*, and *phot1-C* cells were grown as described in the legend of Fig. 1. (**A**) WT was induced with blue (blue bar), red (red bar), or white (white bar) light (30 μmol of photons m^−2^ s^−1^) for 1 hour. (**B**) WT, *phot1*, and *phot1-C* cells were induced as in (A). All transcript levels were normalized to that of the WT in the dark. Error bars represent +SD, *n* ≥ 3. One-way ANOVA was performed. *P < 0.05, **P < 0.01, ***P < 0.001, and ****P < 0.0001. See data S1 for all statistical analysis.

After showing that LL is sufficient to cause substantial accumulation of *LHCSR1*, *LHCSR3.1*, and *PSBS1* mRNA, we tested whether this LL induction was blue- and/or red-light dependent. *LHCSR1*, *LHCSR3.1*, and *PSBS1* transcripts were quantified following exposure of WT cells to low levels of blue, red, and white light (see spectra of light sources in fig. S3). For WT cells, transcripts from the three photoprotective genes increased two to three orders of magnitude relative to control cells when exposed to blue light (30 μmol of photons m^−2^ s^−1^), as shown in Fig. 3 (A and B). A similar level of transcript accumulation was observed in white light (30 μmol of photons m^−2^ s^−1^; Fig. 3A). Red light exposure (30 μmol of photons m^−2^ s^−1^) led to much lower transcript accumulation than in either blue or white light (Fig. 3A, note log scale). Changes in levels of transcripts from the *LHCSR3.2* and *PSBS2* genes in response to blue and red light were similar to those observed for *LHCSR3.1* and *PSBS1*, respectively (fig. S4A).

The similar level of transcript accumulation in blue and white light (Fig. 3A) and the much weaker impact of red light suggest that most of the LL increase in expression of these genes is explained by the impact of the blue light photoreceptor PHOT1. To substantiate this, blue light–dependent transcript accumulation was examined in WT cells, the *phot1* mutant, and the PHOT1-rescued strain (*phot1-C*). The *phot1* mutant used in this analysis was generated by CRISPR-Cas9 editing (*40*) and exhibits a similar phenotype to that of the previously published *phot1* mutant (*17*); there is a loss of the PHOT1 protein, a reduction in the level of the LHCSR3 protein (fig. S5A) and a marked decrease in the capacity of the cells to perform NPQ (fig. S5B). In the *phot1-C* strain, *PHOT1* is ectopically expressed, reaching a transcript level similar to that of WT cells; this strain has a restored capacity to synthesize high levels of LHCSR3 protein and to perform NPQ (fig. S5). As shown in Fig. 3B, the blue light triggered increases in the transcripts from the photoprotective genes were strongly suppressed in the *phot1* mutant and rescued in the *phot1-C* strain. *LHCSR3.1* and *PSBS1* showed very little mRNA accumulation (not statistically significant) in low blue light–exposed *phot1* cells, whereas the *LHCSR1* transcript still exhibited a low but significant level of accumulation in the mutant. Overall, disruption of the *PHOT1* gene led to a ≥96% reduction in accumulation of all photoprotective gene transcripts following exposure to blue light (30 μmol of photons m^−2^ s^−1^) (Fig. 3B).

These results demonstrate that LL-elicited accumulation of the photoprotective transcripts is strongly dependent on blue light photoperception by PHOT1, with potentially a small impact through an alternative photoreceptor (e.g., cryptochromes) and/or a photoreceptor-independent pathway that is responsive to both red and blue light. The PHOT1-independent light effect on accumulation of these transcripts, especially for *LHCSR1*, could also reflect a small impact of electron transport in modulating accumulation of these transcripts, which is supported by the finding that DCMU affects their abundances to a small extent (especially in LL), as noted in Fig. 2 and fig. S4 (B and C); the impact of electron transport was more evident in the *phot1* mutant, as DCMU almost completely repressed *LHCSR1* expression because of the lack of the PHOT1-dependent regulation (fig. S4C).

### UV-B radiation elicits UVR8-dependent, PAR-independent accumulation of mRNA from the photoprotective genes

As shown in Fig. 1A, UV-B light peaks at the same time of the day as PAR, although its increase is delayed relative to PAR and its decrease occurs several hours ahead of the PAR decrease, with very low intensity during the early morning and late afternoon. Furthermore, unlike PAR, UV-B is not diminished much by cloud cover. The role of UV-B radiation on expression of the photoprotective genes was previously investigated, showing that supplementation of very LL (5 μmol of photons m^−2^ s^−1^) with UV-B radiation leads to an increase in accumulation of *LHCSR1*, *LHCSR3.1*, and *PSBS1* transcripts (*7*). Similarly, we observed that UV-B light has an augmenting effect on transcript accumulation when cells were exposed to white light (30 μmol of photons m^−2^ s^−1^; fig. S6). For these studies, we used two WT strains (CC-124 and CC-125) and monitored the kinetics of transcript accumulation following exposure of the cells to LL and LL + UV-B radiation over a 1-hour period. A gradual accumulation of each of the three transcripts was observed, with a significant difference (10- to 20-fold) between LL and LL + UV-B after 1 hour of irradiation (fig. S6).

The kinetics of induction of the target genes in CC-124 were slightly different relative to CC-125; the transcripts reached maximal levels a little more rapidly in CC-124. The maximum difference between the levels of these transcripts measured in LL and LL + UV-B appeared to occur between 30 min and 1 hour. *PSBS1* transcript accumulation appeared to be more strongly elevated in CC-124 by supplementation with UV-B radiation than that of *LHCSR1* or *LHCSR3.1*, especially when measured shortly after the initiation of UV-B exposure (15 min), reaching a maximal level after approximately 30 min, which agrees with previously published data (*7*). Overall, supplementation of LL-maintained cells with UV-B radiation caused a marked (≥10-fold) increase in levels of mRNA from the three photoprotective genes after 15 to 60 min of UV-B exposure.

**Fig. 4.**
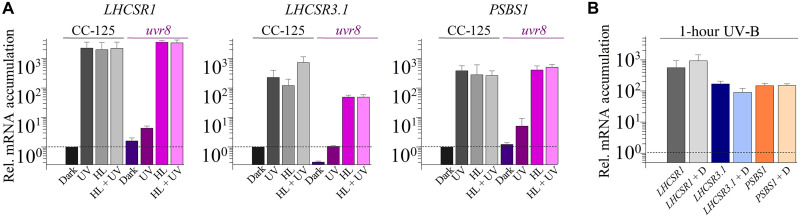
Impact of UV-B radiation on expression of photoprotective genes. (**A**) Changes in levels of *LHCSR* and *PSBS* transcripts after 1 hour of UV-B (UV) radiation, HL, or HL + UV-B radiation. WT CC-125 (gray-black bars) and *uvr8* cells (colored bars) were grown as described in the legend of Fig. 1. Cultures were then divided and exposed to UV-B irradiation (200 μW cm^−2^), HL (480 μmol of photons m^−2^ s^−1^), or HL in the presence of UV-B radiation. (**B**) Cultures were grown as in (A), and all samples were exposed to UV-B radiation in either the absence or presence of 10 μM DCMU (+D). The dashed line across the bar graph indicates the level of transcript in the dark before illumination. All transcript levels in both (A) and (B) were normalized to the transcript level of the WT in the dark. *n* = 3 to 7 + SD. Statistical analyses and *P* values are listed in data S1.

The UV-B radiation used in these experiments (200 μW cm^−2^) corresponds to the maximum intensity observed at noon on a summer day in California (Fig. 1A). Usually, this UV-B level is accompanied by the highest PAR intensity measured during the day, although, at times, much of the PAR can be blocked by cloud cover without strongly affecting UV-B penetrance. To dissect the specific contribution of UV-B light, we examined its effect on gene expression in the presence or absence of high PAR. In addition, mRNA accumulation was measured in both WT cells and the *uvr8* mutant, which is null for the UV-B photoreceptor. As shown in Fig. 4A, UV-B irradiation of WT cells in the absence of PAR elicited an unexpectedly high increase in accumulation of *LHCSR1*, *LHCSR3.1*, and *PSBS1* transcripts. The extent of this increase for the *LHCSR1* and *PSBS1* transcripts was essentially identical to that observed when the cells were exposed to HL (480 μmol of photons m^−2^ s^−1^), with no additional increase in cells simultaneously exposed to HL and UV-B radiation. However, the highest level of *LHCSR3.1* transcript accumulation occurred in cells exposed to both HL and UV-B radiation (increase by an additional ~5-fold with UV-B irradiation). This observation may reflect an inability of the levels of either UV-B or HL radiation alone to fully saturate the induction of *LHCSR3.1*; the inability to saturate the *LHCSR3.1* transcript accumulation at 480 μmol of photons m^−2^ s^−1^ was also observed in Fig. 1 where the level of the *LHCSR3.1* mRNA under the highest irradiation, 960 μmol of photons m^−2^ s^−1^, was elevated by two- to threefold relative to the level at 480 μmol of photons m^−2^ s^−1^. Therefore, we performed the same experiment as in Fig. 4, but the PAR light level used was 960 μmol of photons m^−2^ s^−1^ [very HL (VHL)] (fig. S7A). Similar to the observations presented in Fig. 1, *LHCSR3.1* transcript accumulation in cells exposed to VHL was about twice as high as that of cells exposed to HL (compare Fig. 4A with fig. S7A), with a similar level attained when the cells were only exposed to UV-B radiation. However, transcript accumulation in VHL and HL, both supplemented with UV-B, followed the exact same trend (compare fig. S7A with Fig. 4); the level of the *LHCSR3.1* transcript was identical in UV-B and in VHL, while combining the two light sources led to higher *LHCSR3.1* transcript accumulation (~5-fold). These results suggest that while PAR and UV-B light can reach similar levels and compensate for each other with respect to *LHCSR3.1* mRNA accumulation, only simultaneous exposure to both types of radiation promote maximum transcript accumulation under the PAR levels tested in this work.

We next tested the role of the UVR8 photoreceptor in controlling accumulation of transcripts from the photoprotective genes. The *uvr8* mutant had markedly reduced levels of transcripts from all three of the photoprotective genes relative to WT cells following UV-B exposure (Fig. 4A), indicating that UVR8 is integral to the regulation of these transcripts. For *LHCSR1* and *PSBS1*, the transcript levels attained in HL and HL + UV-B radiation were not affected by the loss of the UVR8 photoreceptor. However, the mutant exhibited slightly lower levels of *LHCSR3.1* mRNA in HL and HL + UV-B radiation; this reduction was three- to fourfold relative to WT cells, although the overall fold induction was comparable in both strains (the dark levels of the *LHCSR3.1* transcript were lower in the *uvr8* mutant); these differences are not statistically significant. Similar results were obtained for the impact of HL and HL + UV-B radiation on the patterns of transcript accumulation for *LHCSR3.2* and *PSBS2* relative to those of *LHCSR3.1* and *PSBS1*, respectively (fig. S7B). In addition, as shown in Fig. 4B, the addition of DCMU had no impact on UV-B–induced expression of these genes, demonstrating that all transcript accumulation during exposure to UV-B is independent of linear PET. Last, these data also suggest that there is a very small (≤1%) UV-B–dependent, UVR8-independent accumulation of the *LHCSR1* and *PSBS* transcripts (Fig. 4A) that could be a consequence of stimulation of the PHOT1 photoreceptor, which has very low absorption in the UV-B region of the spectrum or be triggered by reactive species (RS) generated as a consequence of UV-B radiation–mediated damage (*41*–*43*).

### Light intensity and CO_2_ levels independently affect accumulation of transcripts from the photoprotective genes

CIA5 is a regulatory element that controls acclimation of Chlamydomonas to low CO_2_ conditions [e.g., induction of carbon concentrating mechanism (CCM)] (*44*, *45*). Although *LHCSR3* gene expression has been associated with HL, it was also shown to be highly dependent on the level of CO_2_; transcript levels decreased when the CO_2_ concentration of the culture was elevated (*46*). Furthermore, previous work reported that expression of *LHCSR3* is affected in the *cia5* mutant (*44*).

**Fig. 5.**
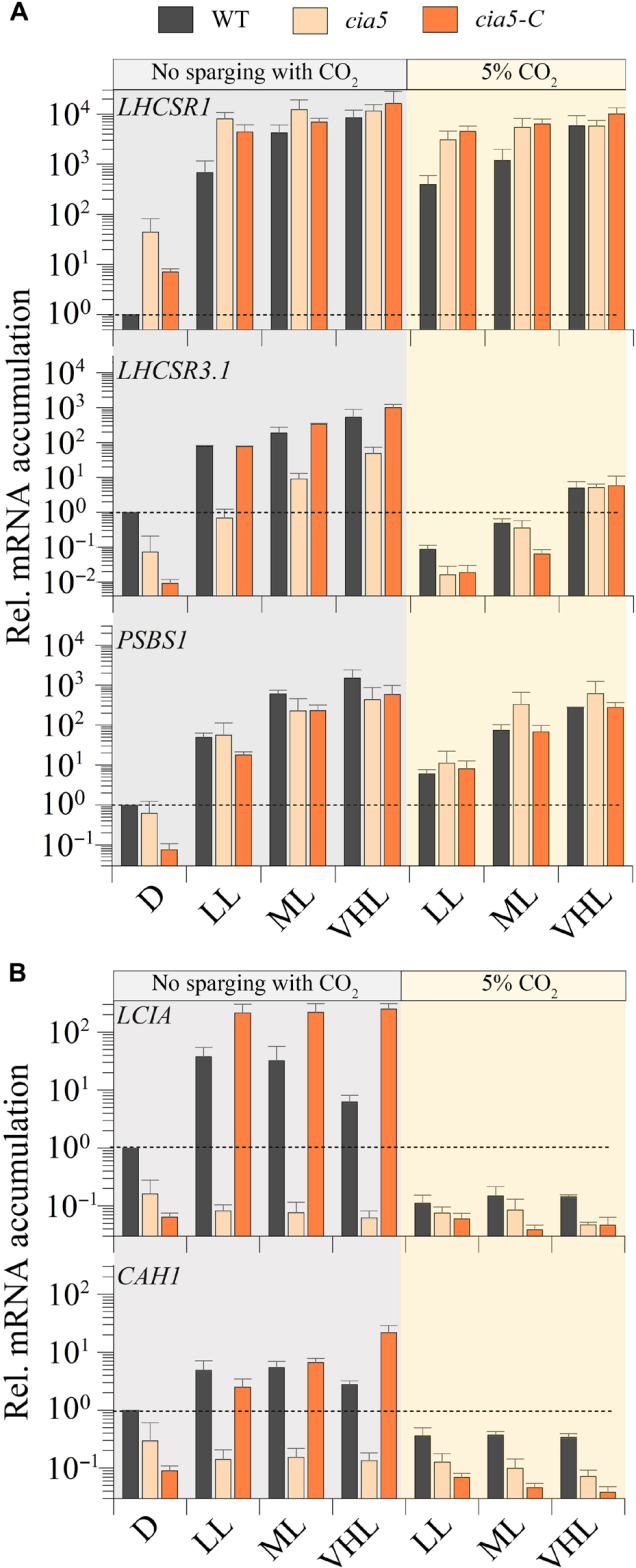
Impact of different light intensities and CO_2_ levels on expression of photoprotective genes. CC-125, *cia5* mutant cells, and the rescued *cia5-C* strains were grown and dark adapted as described in the legend of Fig. 1 and then transferred to HSM or HSM + 5% CO_2_ for 1 hour at LL (30 μmol of photons m^−2^ s^−1^), ML (120 μmol of photons m^−2^ s^−1^), or VHL (1000 μmol of photons m^−2^ s^−1^). Transcript levels were normalized to the level in WT cells before induction (D, dark) (dashed line). *n* = 3 + SD. Statistical analyses and *P* values are listed in data S1.

To study the role of CIA5 in the regulation of the photoprotective genes and determine whether the light- and CO_2_-CIA5–dependent transcriptional regulations of these genes are linked, WT, the *cia5*-null mutant, and a *cia5*-rescued strain (*cia5* mutant with WT *CIA5* gene ectopically expressed; *cia5-C*) were exposed to LL, moderate light (ML; 120 μmol of photons m^−2^ s^−1^), and VHL (1000 μmol of photons m^−2^ s^−1^) at both ambient and high CO_2_ levels, and changes in *LHCSR1*, *LHCSR3.1*, and *PSBS1* transcript levels were analyzed. As shown previously, the absence of CIA5 negatively affected accumulation of the *LHCSR3.1* transcript (*44*, *46*); however, our results also show that VHL intensities can partially compensate for the lack of CIA5 as differences in transcript accumulation were smaller between WT and *cia5* strains when the light intensity was increased (Fig. 5A). The *cia5* mutant exhibited a significant increase in *LHCSR3.1* transcript accumulation at ML and VHL; in VHL, the mRNA accumulation was ~15-fold higher than in LL and >100-fold higher than in the dark. This mRNA accumulation in the mutant supports the idea that light can regulate *LHCSR3.1* expression in a CIA5-independent way. Furthermore, elevated CO_2_ levels strongly suppressed transcript accumulation leading to similar *LHCSR3.1* mRNA levels in both the WT and *cia5* strain, because, under high CO_2_ conditions, there is no requirement/role for CIA5 regulation. Nevertheless, even in high CO_2_, there was still some induction of the *LHCSR3.1* gene (but still very low level of transcript accumulation) at the higher light intensities, again pointing to the participation of a CIA5-independent pathway in *LHCSR3.1* transcriptional regulation. Moreover, *LHCSR3.1* transcript accumulation was also analyzed in WT, *phot1*, and *cia5* in blue LL (fig. S4E), which demonstrated that the mRNA levels in WT and *cia5* showed the same trend in blue as in white LL (Fig. 5). The PHOT1-dependent increase in *LHCSR3.1* transcript abundance was strongly suppressed by high CO_2_ (fig. S4E), which raises the possibility that the PHOT1-dependent regulation might be an indirect effect caused by a reduction in the CO_2_ levels. In contrast to the results obtained for *LHCSR3.1*, CIA5 barely affected *LHCSR1* expression in cells exposed to LL, ML, or VHL in the presence or absence of 5% CO_2_ (Fig. 5), while *PSBS1* transcript accumulation in the absence of CO_2_ supplementation in LL, ML, and VHL was similar in WT, *cia5*, and the *cia5-C*–rescued strain (see below for further discussion).

We performed the same analyses as described above with two CCM genes that were previously shown to be under CIA5 control (*47*). These genes (*CAH1* and *LCIA*) were up-regulated at ambient CO_2_ levels, in the presence of light, and in a CIA5-dependent way in WT cells. Contrary to the results for *LHCSR3.1*, *CAH1*, and *LCIA* transcript accumulation was not strongly affected under all light conditions used in this experiment (LL, ML, and VHL) at ambient levels of CO_2_. Their induction was suppressed in WT cells sparged with 5% CO_2_ (under all light conditions) to very low levels, not observed in the *cia5* mutant at any light intensity, and the phenotype was rescued in the complemented strain, which often exhibited even higher transcript levels than the WT strain (possibly due to overexpression of the ectopic *CIA5* gene; Fig. 5B). These results suggest that CIA5 is absolutely required for expression of *CAH1* and *LCIA* and that any small effects of light may be caused by a reduction in CO_2_ levels. Overall, our results confirm that CIA5 is essential for expression/induction of CCM genes (*CAH1* and *LCIA*) under low CO_2_ conditions, while it appears to function as an enhancer for *LHCSR3.1*, which can still be up-regulated by CIA5-independent light-dependent signals.

### Integration of CO_2_ and UV-B light signals in the transcriptional regulation of the photoprotective genes

**Fig. 6.**
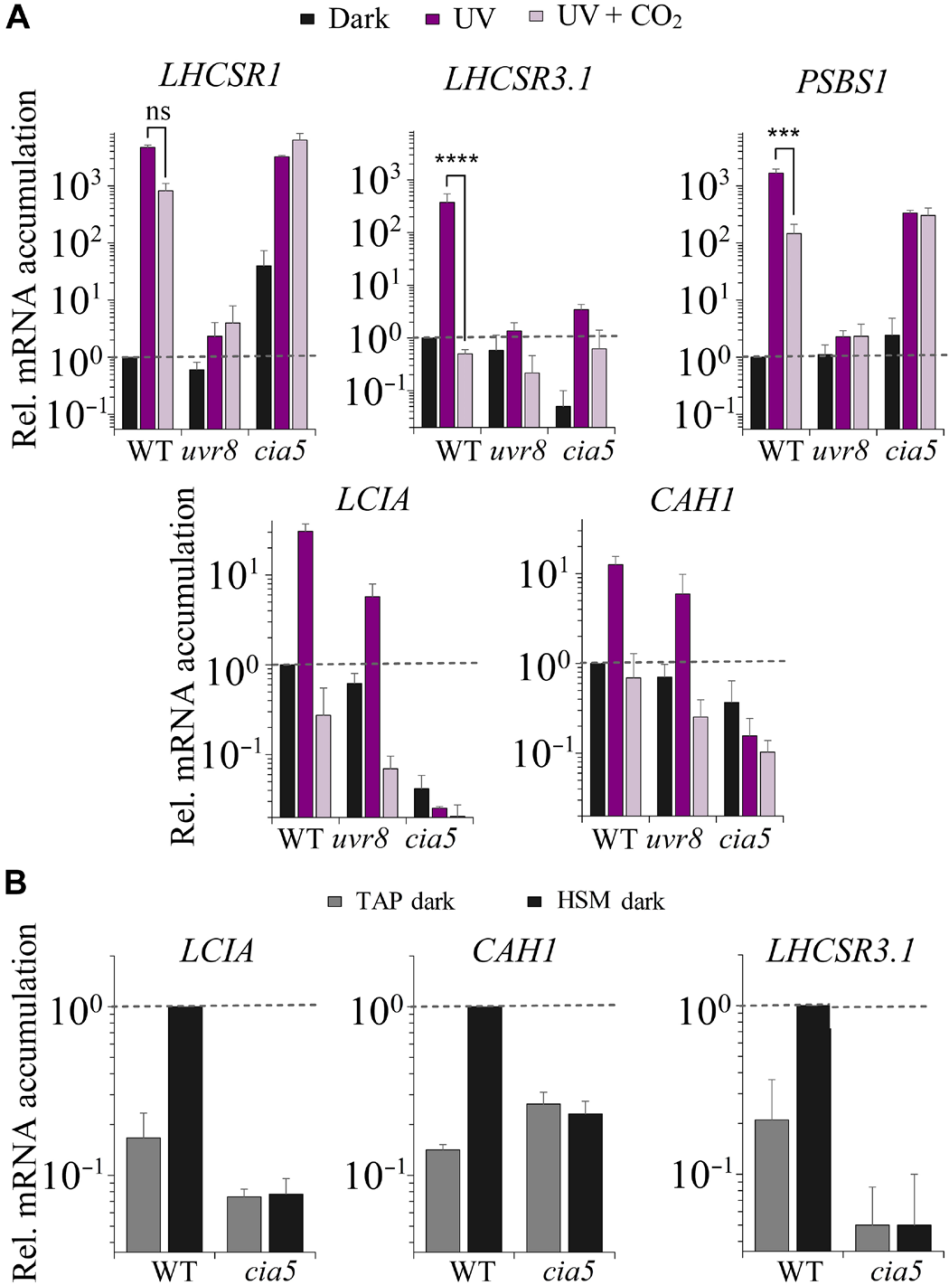
Impact of UV-B radiation, CO_2_, and CIA5 on expression of CCM genes and photoprotective genes. Changes in levels of transcripts from (**A**) the CCM genes *LCIA* and *CAH1* and the photoprotective genes *LHCSR1*, *LHCSR3.1*, and *PSBS* after 1 hour of UV-B radiation (200 μW cm^−2^) in the absence (UV, dark purple bars) or presence (UV + CO_2_, faded purple bars) of 5% CO_2_ in the WT, *uvr8*, and *cia5* strains; and (**B**) from *LCIA*, *CAH1*, and *LHCSR3.1* in the dark after 24 hours in TAP (gray) and after two additional hours following the change of the culture from TAP to HSM (black) in the WT and *cia5* mutant. *n* = 3 + SD. Transcript levels were normalized to the initial level of WT in the dark. Statistical analyses and *P* values are listed in data S1.

We also tested whether the UV-B–elicited responses in transcript abundances for the photoprotective genes were linked to CO_2_ concentrations and CIA5 regulation. We exposed WT, *uvr8*, and *cia5* strains to UV-B light with or without 5% CO_2_ and measured transcript accumulation for the three photoprotective and the two CCM genes previously studied. The UV-B–dependent 400-fold accumulation in *LHCSR3.1* transcript observed in WT cells was completely abolished by sparging the culture with 5% CO_2_ (Fig. 6A). However, although the basal level (dark) of *LHCSR3.1* mRNA was much lower in the *cia5* mutant, the mutant still exhibited an increase in *LHCSR3.1* transcript accumulation following UV-B radiation by almost two orders of magnitude (comparable fold change to WT). These results suggest not only that *LHCSR3.1* up-regulation mediated by UV-B is CIA5-independent but also that CIA5 acts either directly or indirectly to enhance the overall expression of this gene in the dark (also observed in Fig. 5A) and during exposure to UV-B radiation (Fig. 6A). The lower mRNA levels in the *cia5* mutant strain in the dark indicate that *LHCSR3.1* expression was already induced in the dark in a CIA5-dependent way in the WT strain. In our protocol, dark-acclimated cells were transferred from TAP (tris-acetate-phosphate) to acetate free medium (HSM) for 2 hours, still in the dark, before the UV-B exposure. We measured *LHCSR3.1*, as well as *LCIA* and *CAH1* transcript levels, before and after transferring WT and *cia5* cells to HSM and found that this transfer led to an approximate 10-fold increase in *LCIA*, *CAH1*, and *LHCSR3.1* mRNA in WT cells (Fig. 6B). This result can be explained on the basis of the recent findings of Ruiz-Sola and collaborators who demonstrated that changes in CO_2_ availability can activate *LHCSR3* gene expression even in the absence of light in a CIA5-dependent manner (*48*). Here, a similar change in transcript accumulation is found for the CCM-related genes. The transfer of cells from TAP to HSM would cause the CO_2_ levels in the culture to drop [CO_2_ that accumulated in TAP medium (associated with metabolism of acetate) in the dark would decline], which would cause elevated transcription of *LHCSR3*. Under high CO_2_ conditions, CIA5 would not be active and, similar to what we observed in white light, the level of *LHCSR3.1* mRNA would be very low in both WT and *cia5* strains.

The CCM-related genes exhibited an important difference with respect to regulation compared to *LHCSR3.1*; while *LCIA* and *CAH1* mRNA accumulation in cells exposed to UV-B radiation was similar to that observed under white light (compare Fig. 5B and Fig. 6A) and strongly repressed by CO_2_, unlike *LHCSR3.1*, this induction was not UVR8 dependent (it was between 10- and 12-fold for both WT and *uvr8*). Furthermore, unlike *LHCSR3.1*, *LCIA* and *CAH1* transcript accumulation was completely abolished in the *cia5* mutant under all conditions. This indicates that these genes might be exclusively regulated by the CO_2_/CIA5 signaling pathway and suggests that the light-mediated responses (PAR and UV-B) may be indirect, altering the level of CO_2_ and the efficacy of the CO_2_/CIA5 signaling pathway, which is further supported by the finding that these genes in WT cells are induced in the dark upon transfer from TAP to HSM, but not in *cia5* (Fig. 6B). Understanding whether UV-B light can alter the intracellular CO_2_ levels or whether the CCM genes might respond to changes in RS mediated by UV-B radiation will require further investigation.

While *LHCSR1* and *PSBS1* transcripts strongly accumulated upon exposure to UV-B radiation (as shown in Fig. 4), when the cultures were sparged with 5% CO_2_ concomitant with the UV-B exposure, there was only a 5-fold decrease in the *LHCSR1* transcript and a 12-fold decrease in the *PSBS1* transcript, indicating that CO_2_ does not have a strong impact on expression of these genes following a dark-to-light transition. Furthermore, *LHCSR1* and *PSBS1* transcript levels still increased in *cia5* cells induced by UV-B light to a level similar to that observed in WT cells, and this induction was not suppressed by sparging the cultures with high CO_2_. Hence, unlike for the *LHCSR3.1* transcript, the *cia5* mutant only slightly affected *LHCSR1* and *PSBS1* gene expression during UV-B–dependent induction.

## DISCUSSION

The fastest NPQ mechanisms induced upon exposure of Chlamydomonas cells to HL are qE and state transition (qT), which become active in seconds (qE) to minutes (qT) (*49*, *50*). While the transcript levels of qE-related genes are extremely low and the proteins are undetectable in dark-acclimated cells, their induction must anticipate HL exposure to minimize cellular damage. In this work, we analyzed how photosynthetic cells sense and integrate environmental cues to modulate expression of the photoprotective genes *LHCSR1*, *LHCSR3.1*, and *PSBS1*. We show that illumination with even very LL was sufficient to cause substantial accumulation of these photoprotective transcripts in dark-preacclimated cells (Fig. 1B). In nature, this response would allow Chlamydomonas to accumulate these transcripts at dawn, priming the cells for a marked increase in radiation that normally occurs during the first few hours of morning light (Fig. 1A) and compensating for protein degradation that may have occurred overnight. While photoprotective proteins such as LHCSR3 are stable for hours in the dark under optimal and controlled conditions in the laboratory (*51*), this may not occur in the natural environment where cells often experience dynamic, extreme conditions (e.g., nutrient limitation, anoxia, etc.) that might trigger protein turnover.

**Fig. 7.**
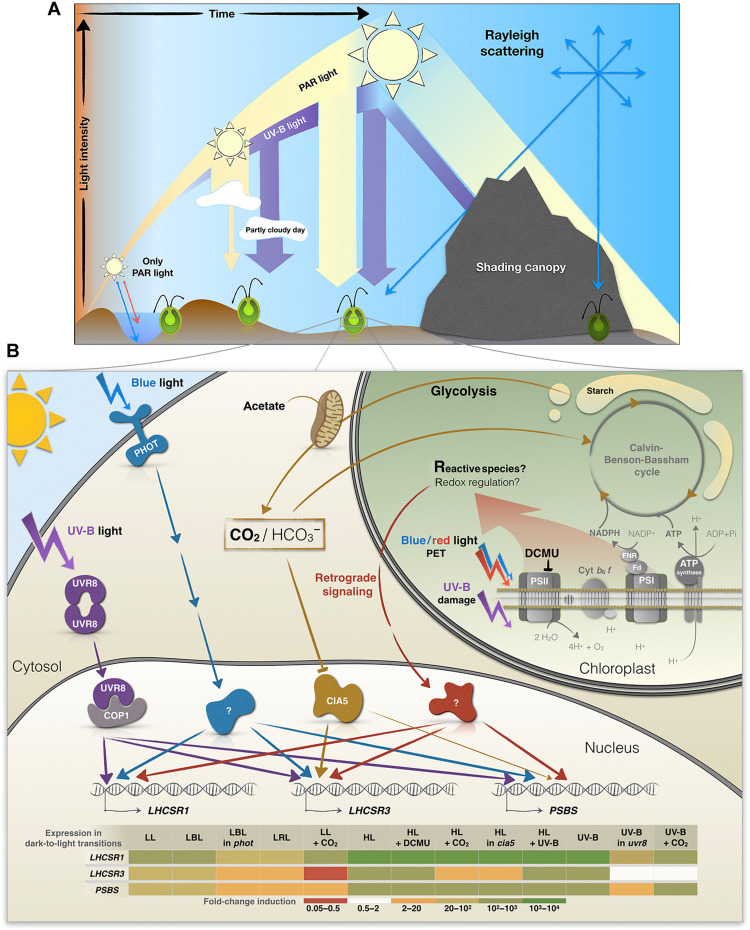
Integration of environmental signals on the expression of the photoprotection-related genes. (**A**) Schematic summary of changing light quality and quantity throughout the day. Blue light reaches deeper levels of the water column, while red light is absorbed near the water surface. In addition, PAR can be strongly reduced by cloud cover, while UV-B radiation might even increase on partly cloudy days. While direct sunlight is shielded/reduced by canopy shading, blue light (and UV light in the case of a plant canopy) reach shaded areas more effectively than other wavelength of PAR through Rayleigh scattering, which increases as the wavelength of light decreases. (**B**) Signals that regulate energy dissipation in Chlamydomonas. Transcription of *LHCSR1*, *LHCSR3*, and *PSBS* is strongly initiated with exposure to a very low amount of white light (5 μmol of photons m^−2^ s^−1^; Fig. 1). This activation is not only strongest for *LHCSR3* but also apparent for *LHCSR1* and *PSBS* and is dependent on the Chlamydomonas blue light–dependent photoreceptor PHOT1. All three transcripts are also partially regulated by PET downstream of PSII and the generation of retrograde signals by HL (red). UV-B radiation directly facilitates monomerization of the UVR8 homodimer, which then binds to COP1 and allows the participation of other factors (not included in the figure) in the transcriptional regulation of the photoprotective genes (purple). UV-B exposure may also lead to the generation of RS in the chloroplast that further triggers signaling events (red). In addition, *LHCSR3* is strongly controlled by CO_2_ levels and CIA5, while *PSBS* may be affected by CO_2_ to a minor extent (orange). Additional discussion of the role of CO_2_ in regulating *LHCSR3* is presented in Ruiz-Sola *et al.* (*48*). The heatmap table summarizes the transcript fold change for each gene in the transition from dark to the indicated conditions. NADP^+^, nicotinamide adenine dinucleotide phosphate; NADPH, reduced form of NADP^+^; ADP, adenosine 5′-diphosphate ; ATP, adenosine 5′-triphosphate.

LL induction of the photoprotective genes was mainly mediated by blue light and the photoreceptor PHOT1 (Figs. 3 and 7B). The proportion of blue light reaching Earth’s surface increases from dawn to mid-day, especially in aquatic environments due to the higher penetrating capacity of shorter wavelengths (*52*). Therefore, the low blue irradiance required to induce photoprotective genes makes this signaling system effective at priming NPQ in both terrestrial and aquatic organisms over the course of the day (Fig. 7A).

Nevertheless, blue light is only one signal of a complex process of control associated with quenching, as suggested by a low but significant increase in *LHCSR1* (and to a lesser extent *LHCSR3.1* and *PSBS1*) mRNA accumulation in the *phot1* mutant following exposure to blue light (Fig. 3B). This PHOT1-independent induction could be mediated by signals generated by chloroplast electron flow and/or by other photoreceptors such as cryptochromes, which can be activated by both blue and red light (*53*) and are required for LHCSX (photoprotective protein) accumulation in diatoms (*54*). In addition, recent work supports a role for chloroplast-generated signals in the regulation of *LHCSR3* expression (*55*). Using DCMU, we confirmed that PSII-dependent electron transport affects the activities of the *LHCSR* promoters (Fig. 2 and figs. S2 and S4, B and C), although the extent of this impact depends on preacclimation conditions (LL or dark) and light intensity. The effect of DCMU on *LHCSR3.1* gene induction was lower in dark-preacclimated cells compared to LL-preacclimated cells (fig. S2). The strong induction observed in dark-preacclimated cells in the presence of DCMU appears to be partially mediated by PHOT1. This photoreceptor would already be activated in LL-preacclimated cells, leading to an increase in “basal” transcript levels of the photoprotective genes (before the HL treatment), with the consequent reduction in the apparent impact of HL on the transcript levels, and an increase in the apparent impact of DCMU on suppressing the HL induction. Moreover, besides differences in the initial PHOT1-dependent preacclimation levels of the photoprotective transcripts, LL-preacclimated cells would have an active PET system already coupled to bicarbonate uptake and CO_2_ fixation, while in dark-preacclimated cells, both uptake and fixation would have to be activated following the preacclimation period. Therefore, in the latter, the initial exposure to HL might generate more RS that could stimulate activation of the photoprotective genes. In contrast, exposure of LL-preacclimated cells to HL in the presence of DCMU would cause an immediate rise in the intracellular CO_2_ concentrations, which, in turn, would cause stronger repression of *LHCSR3.1*.

On the other hand, the slight repression of DCMU on *LHCSR1* occurred only when cells were exposed to LL. This effect was consistently observed in low blue and white light (Fig. 2 and fig. S4B) and when the *phot1* mutant was treated in low blue light (fig. S4C). Why DCMU represses *LHCSR1* only under LL intensities requires further investigation, although some possibilities are discussed below.

Regarding how PET affects expression of the photoprotective genes, previous studies have shown that the redox state of the PQ pool is not relevant for this regulation. Two photosynthesis inhibitors with opposite effects on the PQ pool redox state, DCMU and dibromothymoquinone (DBMIB), both inhibited LHCSR3 protein accumulation (*56*). Other signals generated by PET that affect gene expression include RS and redox signaling, especially as the light intensity increases (noted as retrograde signaling in Fig. 7B). Singlet oxygen, mainly synthesized in the PSII antenna, should be produced at higher concentrations in the presence than in the absence of either DCMU or DBMIB, suggesting that this type of RS most likely does not cause *LHCSR3* or *LHCSR1* induction. Furthermore, DBMIB blocks electron transport by binding at the Q_o_ site of Cyt *b_6_f* (*37*), indicating that the signal required for induction is likely generated downstream of the Q_o_. In addition, RS production in PSI was reported to increase under intense electron flow (HL and excess CO_2_) (*56*). Therefore, DCMU-treated cells would exhibit reduced electron flow to PSI, diminished RS production and, consequently, lower *LHCSR1* and *LHCSR3* expression. Nevertheless, PSI could still accept electrons (at much lower rate) derived from starch breakdown and generate levels of RS that would vary depending on light intensity. In dark-preacclimated cells, upon exposure to light in the presence of DCMU, electron flow through PSI could be enhanced as the lack of photosynthetic O_2_ evolution (because of DCMU) would result in hypoxia and delayed state 2–to–state 1 transition (*57*), elevating cyclic electron flow and, thus, RS generation. This enhanced PSI-dependent RS production, together with a lower intracellular CO_2_ concentration (no CCM induced), could explain the much higher levels of *LHCSR3* mRNA observed in Fig. 2 and fig. S2. The fact that *LHCSR1* responds to DCMU in LL but not in HL, whereas *LHCSR3* seems to be affected at both light intensities could be a consequence of different RS sensitivities. In LL, a reduction of the RS levels would lower the expression of both genes, while in HL, the RS generated even in the presence of DCMU would be enough to induce maximum expression of *LHCSR1*. Previous findings (*58*) and our data suggest that transcription of *LHCSR1* may be more sensitive to RS than *LHCSR3*, as the latter needs higher light intensities to reach maximal expression levels (Figs. 1 and 4).

In addition to RS production by PSI, it has been recently reported that PSII isolated from plants can generate superoxide and that this production is inhibited by bicarbonate (*59*). Therefore, our results could also be explained by higher superoxide production in PSII in dark-acclimated cells than in those preacclimated in LL. However, this PSII-located superoxide production has not been demonstrated in Chlamydomonas and further investigation will be required.

While PHOT1 may have an essential role in inducing NPQ-related genes in “morning” LL (blue pathway in Fig. 7B), the chloroplast-generated signals (red pathway in Fig. 7B) would modulate this expression according to light intensity. The higher the light intensity, the higher the rate of CO_2_ uptake (until saturation) and the lower the level of internal CO_2_ (brown pathway in Fig. 7B), while, at the same time, more PET-related signals would be generated; the highest level of transcript accumulation (Fig. 1B) would reflect both a diminished CO_2_ concentration and elevated production of photosynthetically generated RS.

Several hours after dawn, the solar spectrum is progressively enriched in UV-B (Fig. 1A). Our results indicate that the levels of the *LHCSR1*, *LHCSR3*, and *PSBS* transcripts in cells solely exposed to UV-B light were similar to those of cells incubated in HL or HL + UV-B, except for *LHCSR3* that exhibited some increased induction upon exposure to HL + UV-B radiation (Fig. 4). The saturating or near-saturating response mediated by UV-B light in the absence of PAR was strongly diminished in the *uvr8* mutant, although residual, very low-level induction was still observed for the three genes (Fig. 4). This residual induction may be the consequence of residual PHOT1 stimulation by UV-B light or enhanced RS production upon UV-B exposure, which occurs in both animal and plant cells (*41*–*43*, *60*, *61*).

The UV-B–dependent, PAR-independent pathway activating photoprotective genes may not represent an advantage under a clear sky, where cells would experience HL before exposure to high UV-B radiation (Fig. 1A). However, UV-B radiation might have a role in maintaining maximal promoter activity for the photoprotective genes during a long period of exposure to HL because the levels of these transcripts peak within the first hour of HL exposure, with a significant decline over longer periods (*6*). UV-B perception would also help organisms sense the time of day and when the light intensity is likely to be at its highest, although the organism may not be experiencing excess PAR. This situation is common under conditions of cloud cover (Fig. 1A). Under partly cloudy skies, UV-B radiation may increase in intensity by ∼25% relative to clear skies (*62*). Thus, UV-B perception would prime the system for triggering NPQ even when PAR intensities vary (Fig. 7A). Preacclimation in LL + UV-B radiation was previously shown to improve survival following a sudden exposure of cultures to HL (1000 μmol of photons m^−2^ s^−1^); this protection is mainly mediated by LHCSR1 and, to a lesser extent, by LHCSR3 (*7*).

In addition to light intensity and quality, carbon availability regulates NPQ. The *LHCSR3* transcript accumulates when inorganic carbon levels are low. This induction is mediated by CIA5, the main regulatory factor that controls genes associated with the CCM (*44*, *63*). PSBS protein levels are also elevated more in minimal (air levels of CO_2_) than in TAP medium (17 mM acetate) (*4*); internal CO_2_ levels would increase in the presence of acetate (*48*). In contrast, *LHCSR1* transcript levels accumulated in the presence of high CO_2_, especially upon exposure to HL (*64*). Our results confirm that *LHCSR3* is markedly repressed by high CO_2_ and that its induction under limiting inorganic carbon conditions is strongly regulated by CIA5 (Fig. 5). However, we also demonstrated that light affects *LHCSR3* expression independently of CIA5; the *cia5* mutant responded to different light intensities during exposure to both low and high CO_2_ (Fig. 5), although the absolute levels attained under high CO_2_ were much lower. The higher levels of the *LHCSR3* transcript observed in the *cia5* mutant incubated under low relative to high CO_2_ could result from higher RS accumulation as a consequence of CO_2_ depletion and an increased use of O_2_ as a terminal electron acceptor (*56*). Overall, a variety of signals appear to converge on the control of *LHCSR3* activity to allow for increased survival in the natural environment (Fig. 7).

Our work suggests that CIA5 acts as an enhancer, a positive regulatory element that potentiates transcriptional regulation in conjunction with other regulatory elements (Fig. 7B). We observed that *LHCSR3* was still induced by blue light (fig. S5) and UV-B radiation (Fig. 6) in the *cia5* mutant, although both the basal and induced levels of expression were much lower than in WT cells (but the fold change was similar). The lower *LHCSR3* mRNA levels present in dark-acclimated *cia5* mutant relative to WT cells in minimal medium are in line with the work by Ruiz-Sola *et al.* (*48*), which has demonstrated that CIA5-dependent *LHCSR3* induction also occurred in total darkness when the availability of inorganic carbon becomes very low (Fig. 6B).

The light signal that regulates *LHCSR3* in the *cia5* mutant did not affect expression of the CCM genes (*LCIA* and *CAH1*; Fig. 5), suggesting that the CCM genes may strictly respond to inorganic carbon availability through CIA5-dependent activation. The light effect traditionally ascribed to regulation of the CCM genes (*63*) may exclusively be associated with changes in intracellular inorganic carbon levels resulting from differences in the rate of CO_2_ fixation at the different light intensities.

In contrast, high CO_2_ caused almost no changes (statistically insignificant repression) in *LHCSR1* transcript accumulation in both WT and the *cia5* mutant (Figs. 5 and 6). However, the response of *LHCSR1* to LL and ML was significantly different in WT and *cia5* mutant. *cia5* cells were already able to attain maximal levels of *LHCSR1* transcript at these two light intensities, potentially as a consequence of their reduced ability to concentrate CO_2_ after the dark preacclimation (the basal CCM level is lower in the mutant than in WT; Figs. 5B and 6B), leading to higher RS generation in the mutant after light exposure. Overall, our results suggest that there is an important role for chloroplast-generated signals (i.e., RS) in activation of the photoprotective genes, especially *LHCSR1*.

The *PSBS1* gene exhibited a significant induction in the absence of CO_2_, although this effect was only slightly regulated by CIA5, especially at ML and HL (Fig. 5). However, the CIA5-dependent regulation was more pronounced when WT and *cia5* mutant cells were exposed to UV-B light under low CO_2_ (Fig. 6A). Both *PSBS* genes (*PSBS1*/*2*) contain two enhancer elements (EEC motifs) in their promoter (*4*) that are conserved in low CO_2_-responsive genes, such as *LHCSR3* (*39*), and various CCM genes (*65*, *66*). Increases in the levels of the LHCSR3 and PSBS proteins in response to low CO_2_ have been ascribed to those EEC motifs (*4*). PSBS protein synthesis is induced in HL, but it is rapidly degraded, except when the cells are incubated under low CO_2_ levels (*4*, *6*). The lower induction at LL intensities compared to *LHCSR1* and *LHCSR3* (Fig. 1), the total lack of repression when PET is blocked by DCMU (Fig. 2), the strong transcript (Fig. 4) and protein induction in the presence of UV-B radiation (*7*), and the regulation of transcript and protein accumulation under low CO_2_ conditions (Fig. 5) suggest that this protein may be required under extreme conditions when the light intensity is maximal and cells are experiencing photoinhibition.

Together, our data highlight the complex, multilayered, and finely tuned regulatory network that controls expression of *LHCSR1*, *LHCSR3*, and *PSBS* genes during a dark-to-light transition and allow cells to acclimate and anticipate HL stress. This intricate regulation includes inputs from blue- and UV-B–light photoreceptors, photosynthetic electron flow (e.g., redox and RS), and CO_2_ levels (primarily through CIA5). These inputs may be independent, interactive, integrative, and compensatory, allowing for optimization of expression in a highly dynamic light environment over the course of the day (Fig. 7). This regulatory complexity might be especially relevant in microalgae such as Chlamydomonas, which are found in diverse habitats including fresh and marine waters, agricultural lands, forests, deserts, snow, and even in the air at altitudes of 1100 m (*67*). Further studies into posttranscriptional regulation of *LHCSR1*, *LHCSR3*, and *PSBS* (transcript stability, translation efficiency, protein stability, turnover, and modification) under different light and atmospheric conditions over the diel cycle will provide additional critical insights into the integrated regulation that modulates photoprotection in nature.

## MATERIALS AND METHODS

### Chlamydomonas strains

The *C. reinhardtii* strains used in this study were WT CC-125 mt^+^ and CC-124 mt^+^ (137c), *phot1* (CC-5392), *uvr8* (CC-5442), *cia5* (CC-2702), the *phot1*-rescued strain, designated *phot1-C*, and the *cia5-*rescued strain, designated *cia5-C*. The *phot1* mutant was engineered by CRISPR-CAS9 inactivation (*40*), and *cia5-C* is described in (*48*). For the complementation of *phot1*, resulting in the *pho1t-C* strain, a 2.25-kb fragment containing the *PHOT1* coding DNA sequence was amplified by polymerase chain reaction (PCR) with KOD hot start DNA polymerase (Novagen) using *PHOT1* forward and *PHOT1* reverse primers (table S1), gel-purified, and cloned into phk330 (*67*) using the Bam HI and Eco RI restriction sites for expression under control of *HSP70/RBC* hybrid promoter. Junctions and inserts were sequenced, and constructs were linearized by Kpn I before transformation into the *phot1* mutant. Linearized plasmid (11 ng kb^−1^) (*68*) was mixed with 400 μl of 1.0 × 10^7^ cells ml^−1^ and electroporated in a volume of 120 μl in a 2-mm-gap electrocuvette using a NEPA21 square-pulse electroporator (NEPA GENE, Japan). The electroporation parameters were set as follows: poring pulse (300 V, 8-ms length, 50-ms interval, one pulse, 40% decay rate, and +polarity), transfer pulse (20 V, 50-ms length, 50-ms interval, five pulses, 40% decay rate, and ±polarity). Transformants were selected on solid agar plates containing zeocin (7.5 μg ml^−1^) and screened on the basis of their NPQ capacity using the following protocol: Transformants grown in liquid TAP medium for 3 days in 96-well transparent microplates were shifted to HSM medium and exposed to 300 μmol of photons m^−2^ s^−1^ for 4 hours before measuring NPQ using a Maxi-Imaging PAM fluorometer (see Chlorophyll fluorescence analysis in the Supplementary Text). Colonies with WT levels of NPQ were chosen as putative complemented strains. This was further confirmed by Western blot analyses using anti-PHOT antiserum (LOV1 domain) as previously described (*69*).

### Growth conditions and induction treatments

Cells were grown to mid-exponential phase [chlorophyll (chl), ~10 μg ml^−1^] at 23°C under continuous white light-emitting diode light (30 μmol of photons m^−2^ s^−1^) with shaking at 130 rpm in 50 ml of TAP medium (Harris 2001) in 250-ml flasks. The spectra of the light sources in the growth chambers are shown in fig. S7, with the spectrum of sunlight shown for comparison. Before experimental treatments, the cells were adjusted in TAP medium to a chl concentration of 10 μg ml^−1^ and acclimated in the dark for 24 hours to lower the levels of the *LHCSR1*, *LHCSR3.1*, *LHCSR3.2*, *PSBS1*, and *PBS2* transcripts. Cells were then harvested by centrifugation (3000*g* for 1.5 min) at 23°C, washed once at room temperature with minimum medium (HSM), then resuspended in HSM, and kept shaking for two additional hours in the dark (to further lower transcript levels). After various treatments, described in Results, the cells were harvested by centrifugation (3000*g* for 1.5 min), flash-frozen with liquid nitrogen, and stored at −80°C until the RNA was extracted. UV-B radiation at a level present in natural sunlight at midday (200 μW cm^−2^) was from a Philips TL20W/01RS narrowband UV-B tube with half maximal transmission at 311 nm. Control samples were maintained under a UV-B–protective plexiglass filter. Experiments using LL (30 μmol of photons m^−2^ s^−1^), HL (480 μmol of photons m^−2^ s^−1^), VHL (960 or 1000 μmol of photons m^−2^ s^−1^) and stepped light levels (from 5 to 960 μmol of photons m^−2^ s^−1^) were as described in the figure legends and the growth conditions section, while exposure to blue (450-nm peak) and red (660-nm peak) light (fig. S3) was in a HiPoint plant growth chamber (FH-1200). To suppress photosynthetic electron flow, DCMU was added to cultures (to 10 μM) in the dark immediately before placing them under the various conditions of illumination.

### Evaluating photosynthetically active and UV-B radiation over the diel cycle

PAR and UV-B intensities were measured during 1 week in July in California, from sunrise to sunset, using a LI-250A Light Meter (LI-COR) and an Inc Solarmeter Model 6.2 (Solar Light Company), respectively. The curves in Fig. 1A show the intensity (in micromoles of photons per square meter per second, also designated μE) of PAR and the UV-B radiation (power density in microwatts per square centimeter) over the course of a representative day from 6:00 a.m. to 8:00 p.m.

### RNA extraction and quantitative reverse transcription PCR

Total RNA was isolated using a phenol/chloroform-based protocol (*70*). Residual DNA was removed by TURBO deoxyribonuclease (Thermo Fisher Scientific), and cDNA was synthesized by reverse transcription of 1 μg of isolated total RNA using the iScript Reverse Transcription Supermix (Bio-Rad) in a 20-μl reaction volume. cDNA was diluted by a factor of 2.5, and then 1 μl of the resulting 50 μl (a total of ~20 ng cDNA) was served as the template in a 20-μl reverse transcription PCR reaction. Real-time PCR was performed with the SensiFast SYBR No-Rox Kit (Bioline) in a Roche Light Cycler 480 as described by the manufacturer. A two-step cycling condition was used (95°C for 2 min, 40 cycles of 95°C for 5 s, and 60°C for 30 s) with the fluorescence yield quantified at the end of each cycle. The *CBLP* gene served as the housekeeping control and relative fold differences were calculated on the basis of the Δ*C*_t_ method (2^−(*C*_t_ target gene − *C*_t_ *CBPL*)^) (*71*–*73*). The primer sequences for transcript quantification are displayed in table S1; specific primer pairs were used to distinguish *LHCSR3.1* and *LHCSR3.2* transcripts and *PSBS1* and *PSBS2* transcripts.

### Statistics

Statistical analysis of the data was performed with GraphPad PRISM8 software (8.4.1) with one- or two-way analysis of variance (ANOVA) using Tukey’s post hoc test or uncorrected Fisher’s least significant difference. The significance of differences between treatments are given as ANOVA-derived *P* values that are depicted in the figures as *, **, or ***, representing values of <0.05, <0.005, and <0.001, respectively.

## Notes

View/request a protocol for this paper from *Bio-protocol*.
