# Supplementary material for: Transcriptional regulation of photoprotection in dark-to-light transition—More than just a matter of excess light energy
Source: Sci Adv. 2022 Jun 3;8(22):eabn1832. doi: 10.1126/sciadv.abn1832 (PMC9166400; doi:10.1126/sciadv.abn1832)
Supplement: Supplementary file 1 — Supplementary text Figs. S1 to S7 Table S1 [file sciadv.abn1832_sm.pdf]

Science Advances
AAAS

Supplementary Materials for

Transcriptional regulation of photoprotection in dark-to-light transition—More than just a matter of excess light energy

Petra Redekop *et al.*

Corresponding author: Emanuel Sanz-Luque, q92salue@uco.es; Petra Redekop, predekop@carnegiescience.edu

Sci. Adv. **8**, eabn1832 (2022)
DOI: 10.1126/sciadv.abn1832

The PDF file includes:

Supplementary text
Figs. S1 to S7
Table S1
Legends for data S1 and S2

Other Supplementary Material for this manuscript includes the following:

Data S1 and S2

Supplementary Text

Supplementary Methods

Protein analysis.

Protein samples of whole cell extracts (0.5 µg chl) were loaded on Mini-PROTEAN TGX, 4-20% Biorad precast gels and blotted onto nitrocellulose membranes. Antisera against LHCSR3 and ATPB were from Agrisera (Vännäs, Sweden) while the PHOT1 antiserum (LOV1 domain) was previously described (*77*). ATPB was used as a loading control. The secondary anti-rabbit antibodies were conjugated to horseradish peroxidase. Immunoblots were developed with the ECL detection reagent and images were obtained using a CCD imager (ChemiDoc MP System, Bio-Rad).

Chlorophyll fluorescence analysis.

Fluorescence-based measurements of photosynthetic parameters were performed with a Maxi-Imaging PAM fluorometer (Heinz Walz GmbH, Effeltrich, Germany). NPQ was calculated using the equation (Fm-Fm')/Fm'. Fm and Fm' are maximum fluorescence yields after dark preincubation and in steady state light, respectively, measured using a saturating light pulse. Before assaying NPQ, the cells were exposed to high intensity actinic light (600 µmol photons m^{-2} s^{-1}) for 4 h to induce *LHCSR3* and then dark acclimated with vigorous shaking for 20 min.

Fig. S1

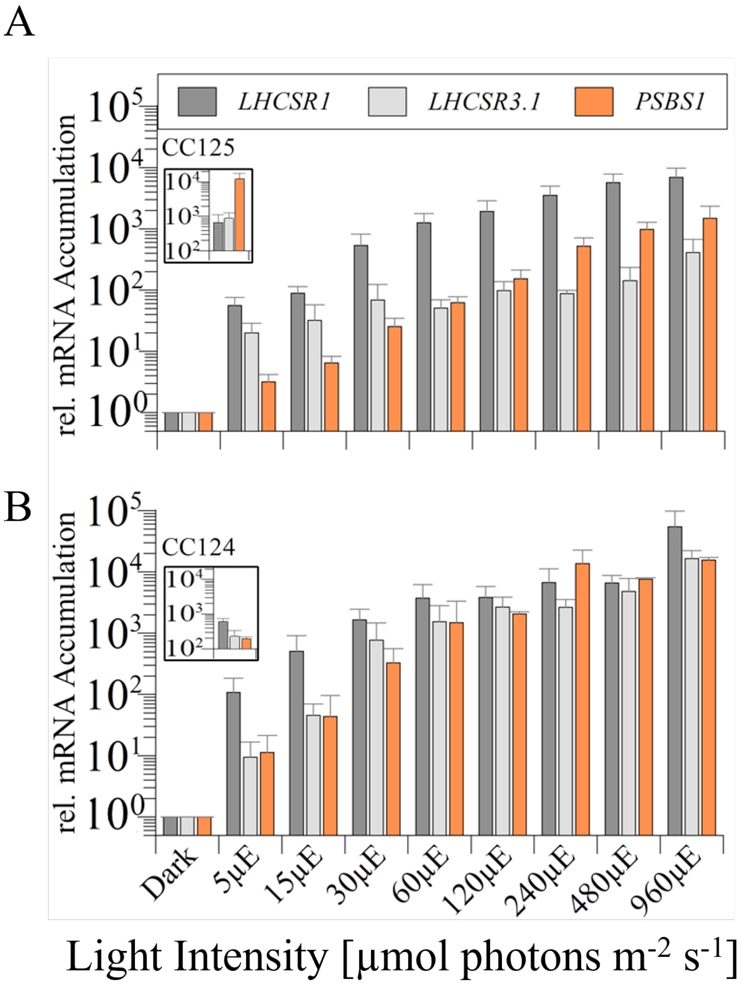

Supplementary Fig. 1. Changes in levels of *LHCSR* and *PSBS* transcripts in CC125 and CC124 after one hour of irradiation at indicated light intensities. *LHCSR1*, *LHCSR3.1* and *PSBS1* transcripts levels were quantified following a 1 h incubation at different light intensities. The **A**) CC-125 (replotted from **Figure 1**) and **B**) CC-124 strains were grown in TAP at LL (30 µmol photons m^{-2} s^{-1}) and then transferred for 24 h to TAP in the dark. Following this incubation, the cells were transferred to HSM (photoautotrophic conditions) and kept for two additional hours in the dark prior to a 1-h light exposure at each of the indicated intensities. Values for each of the three transcripts were normalized to the dark value. The insets in each panel show the initial levels of transcripts determined for cells in the dark before the 1 h treatment period, without normalizing the data. n=3+SD. Statistical analyses and P-values are listed in **Supplementary Data Set 1.**

Fig. S2

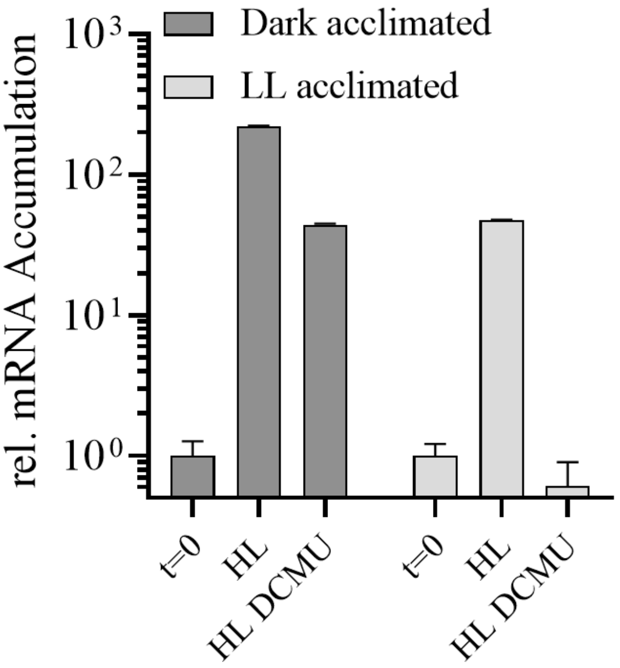

Supplementary Fig. 2. DCMU effect on *LHCSR3.1* mRNA accumulation in cells previously acclimated to either dark or low light. CC-125 cells were acclimated in HSM overnight in darkness or LL (15 µmol photons m^{-2} s^{-1}). The next morning the samples were taken for t=0 and cells were exposed to HL (300 µmol photons m^{-2} s^{-1}) for 1 h in the presence or absence of 40 µM DCMU. n=3+SD. See **Supplementary Data Set 1** for all statistical analysis.

Fig. S3

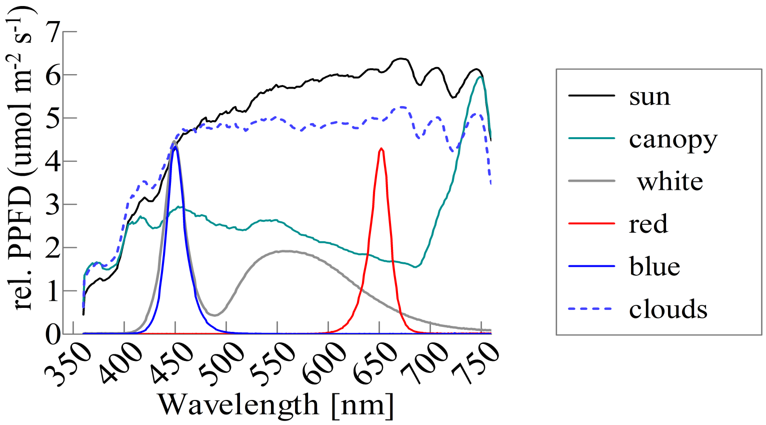

Supplementary Fig. 3. Light spectra of sun light under full sun and cloudy conditions, below a plant canopy, and of the different LEDs used in this study. Spectra of white, red, and blue LEDs used for cell treatment in the growth chambers compared to the spectrum for sunlight, which was measured during the afternoon in full sun and in shade beneath a tree (5:00 PM, California, 2020). Cells were grown in white LED light (grey line). The white LED within the growth chamber is 6000 K, which is highly similar to sunlight/daylight LEDs (6500 K), although the sunlight spectrum is more evenly distributed over the PAR spectrum (400-760 nm).

Fig. S4

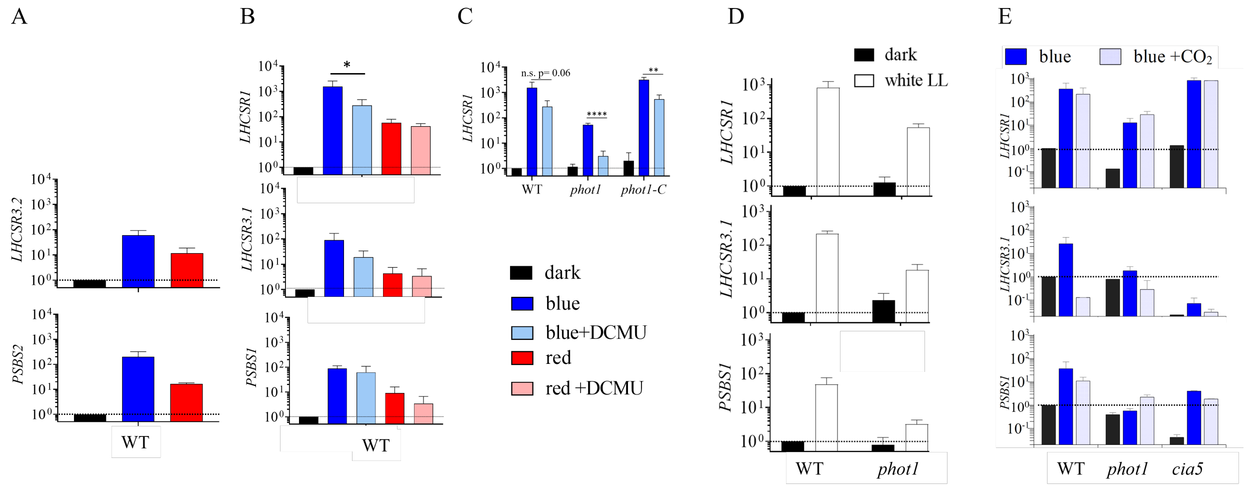

Supplementary Fig. 4. The effects of light quality, electron flow and CO_2 on accumulation of transcripts encoded by the photoprotective genes. A) Induction of *LHCSR3.2* and *PSBS2* genes following 1 h of blue and red light. **B**) Induction of the *LHCSR1*, *LHCSR3.1* and *PSBS1*genes after 1 h of blue or red light in the presence or absence of DCMU (10 µM). **C**) Induction of the *LHCSR1* in WT, *phot1* and *phot1-C* after 1 h of blue light in the presence or absence of DCMU (10 µM). **D**) Induction of *LHCSR1*, *LHCSR3.1* and *PSBS1* genes in WT and the *phot1* mutant for 1 h in LL. **E**) Induction of *LHCSR1*, *LHCSR3.1* and *PSBS1* genes in WT and the *phot1* and *cia5* mutants for 1 h in blue light in the presence or absence of 5% CO_2. CC-125 WT, *phot1* and *cia5* cells were grown as described in the legend of Figure 1 before the various treatments. The light intensities were 30 µmol photons m^{-2} s^{-1} white (white bar), blue (blue bar) or red (red bar) light. Error bars represent +SD, n≥3. One-way ANOVA was performed. *$p<0.05$, **$p<0.01$, ***$p<0.001$, and ****$p<0.0001$. See **Supplementary Data Set 1** for all statistical analysis.

Fig. S5

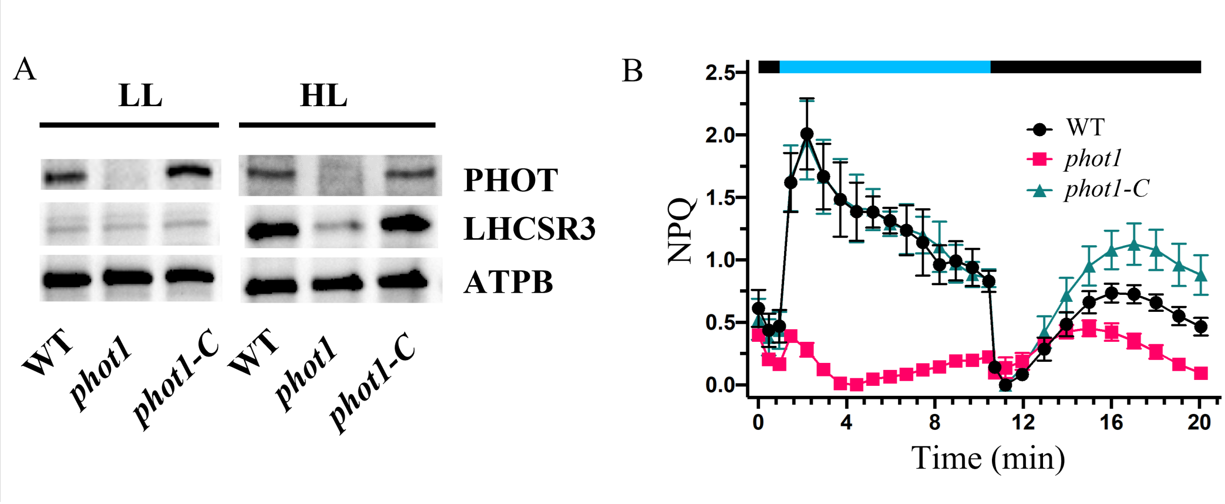

Supplementary Fig. 5. Phenotypes of new *phot1* mutant and the *phot1-C* complemented strain. A) Immunoblot blot analyses of WT, *phot1* and *phot1-C* acclimated to LL (15 µmol photons m^{-2} s^{-1}) and after exposure to HL (300 µmol photons m^{-2} s^{-1}) for 4 h; the proteins examined were PHOT, ATPB and LHCSR3; ATPB served as loading control. **B**) Kinetics of NPQ for WT, *phot* and *phot1-C* strains after exposure to HL for 4 h. NPQ was recorded over 10 min of illumination at 600 µmol photons m^{-2} s^{-1} of blue light (blue bar), followed by 10 min of darkness (black bar), during which relaxation of NPQ was monitored. Values plotted are the means of nine replicates +/- SD (three biological replicates each one measured at three technical replicates). Before the NPQ measurements the cells were shaken in the dark for 20 min. The phenotypes of these strains are in accord with what has been previously reported for similar strains (*17*).

Fig. S6

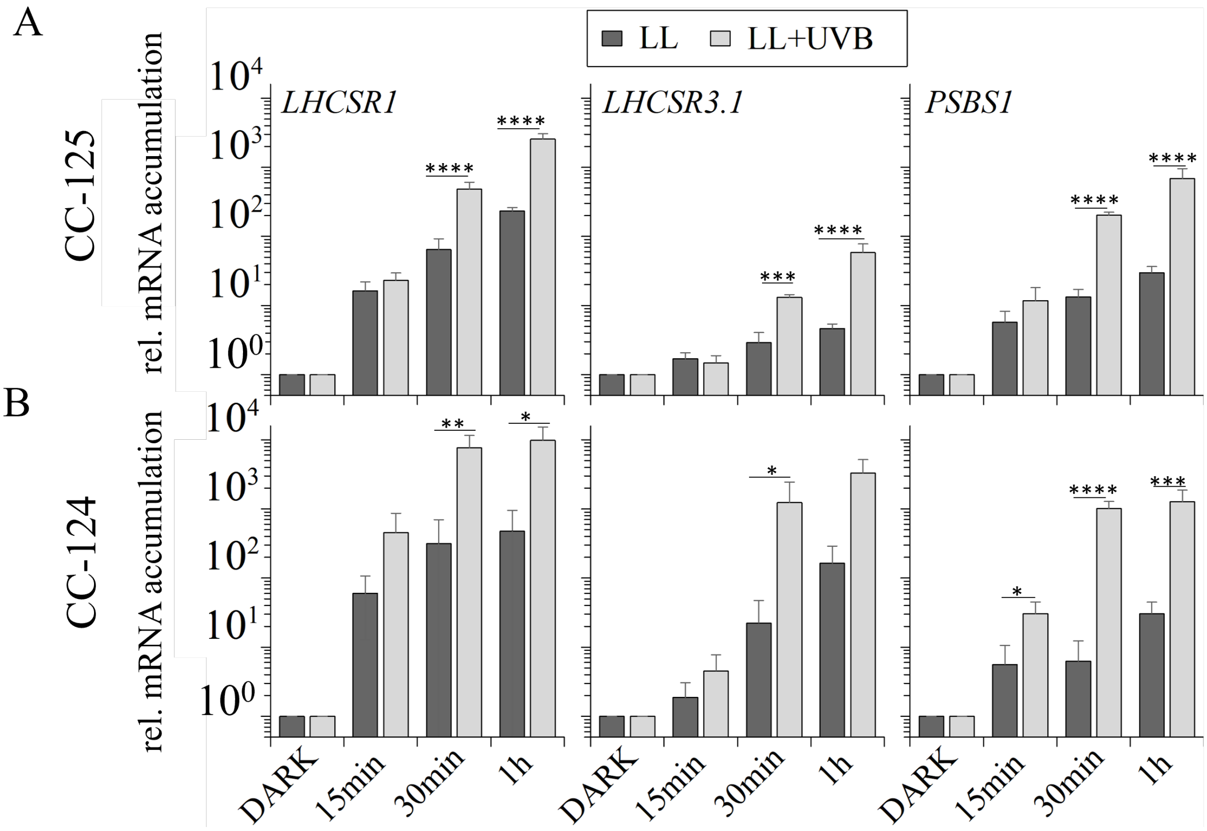

Supplementary Fig. 6. Induction of *LHCSR* and *PSBS* genes at various times in low light in the presence or absence of UV-B irradiation. **A**) WT CC-125 or **B**) WT CC-124 cells were grown as described in the legend of **Figure 1** prior to exposure to LL (30 µmol photons m^{-2} s^{-1}) or LL+UV-B (30 µmol photons m^{-2} s^{-1} PAR plus 200 µW/cm^2 UV-B) for up to 1 h. Error bars represent +SD, n≥3. One-way ANOVA was performed. *p<0.05, **p<0.01, ***p<0.001, and ****p<0.0001. See **Supplementary Data Set 1** for all statistical analysis.

Fig. S7

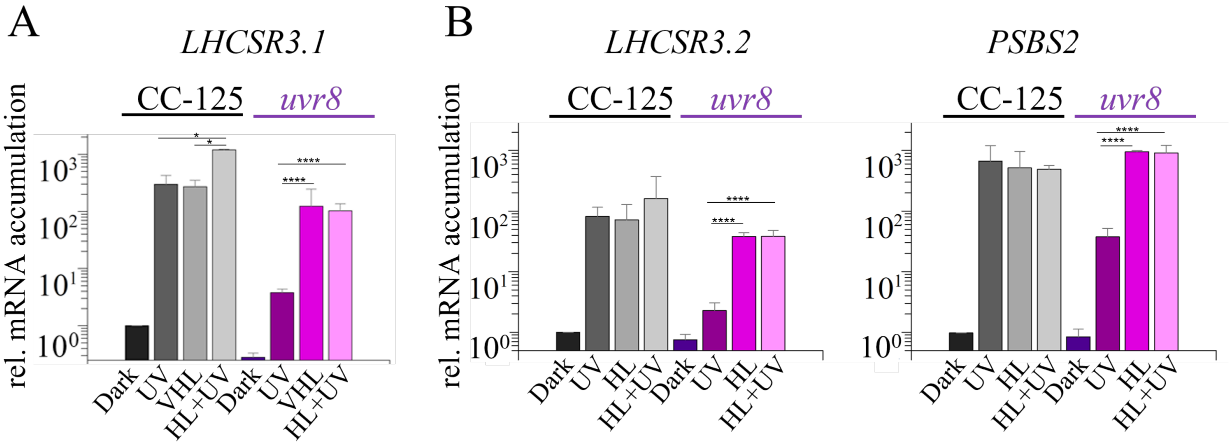

Supplementary Figure 7. Impact of UV-B radiation on expression of other photoprotective genes. Changes in levels of *LHCSR3.1* transcript 1 h after transfer to darkness (Dark), UV-B (UV; 200 µW/cm^2) radiation, VHL (1000 µmol photons m^{-2} s^{-1}), or VHL + UV-B radiation. **B**) *LHCSR3.2* and *PSBS2* after 1 h of darkness, UV-B (UV) radiation, HL (480 µmol photons m^{-2} s^{-1}), or HL+UV-B radiation. WT CC-125 (grey-black bars) and *uvr8* cells (coloured bars) were grown as described in the legend of **Figure 1**. Cultures were grown as described in the legend of Figure 1, divided and either kept in the dark or subjected to the various light treatments described. Error bars represent +SD, n≥3. One-way ANOVA was performed. **p*<0.05 and *****p*<0.0001. See **Supplementary Data Set 1** for all statistical analysis.

Table S1

Primer	Sequence
**LHCSR1 FW**	CTGAGAGGGCCAAAGAATAAGAGC
**LHCSR1 RV**	CCATGAGAGGGGGAAGATACGA
**LHCSR3.1 FW**	CACAACACCTTGATGCGAGATG
**LHCSR3.1 RV**	CCGTGTCTTGTCAGTCCCTG
**LHCSR3.2 FW**	TGTGAGGCACTCTGGTGAAG
**LHCSR3.2 RV**	CGCCTGTTGTCACCATCTTA
**PSBS1 FW**	CCGTGTATTGGAACTCCGTAGG
**PSBS1 RV**	TCCTATCCTTCGCTCTCTTGTG
**PSBS2 FW**	CACTTGTTAGGCAGGTCGAAAG
**PSBS2RV**	CTATCTCTCGCTCTCTCTGCAC
**CBLP FW**	CAAGATCTGGGACCTGGAGAGC
**CBLP RV**	CTGGGCATTTACAGGGAGTGG
**PHOT1 FW**	CCCGGATCCATGGCAGGGGTGC; underlined BamHI site
**PHOT1 RV**	TAGAATTCTCAGTAGTTGTCGAACGCCG; underlined EcoRI site

Supplementary Table S1. Sequence of the primers used in this study.

Data S1. Statistical analysis and P-values of all the qRT-PCR experiments shown in this study (separated file).

Data S2. Raw data used in this manuscript (separate file).
